# 6.M. Workshop: Models of care in prison: addressing infectious diseases during and after the pandemic in EU/EEA

**DOI:** 10.1093/eurpub/ckac129.384

**Published:** 2022-10-25

**Authors:** Organised by: EMCDDA, WHO, UNIPI, EUPHA-PHPP, EUPHA-IDC, Chair persons: Sara Mazzilli (Italy), Sofia Ribeiro (EUPHA-PHPP)

## Abstract

According to the latest data, in 2019 about 497,000 people were held in prison on any given day in the EU. However, the number of people who pass through European prisons each year is considerably higher. Due to infrastructural and population characteristics, individuals in contact with the criminal justice system face multiple and complex health care issues, including a higher prevalence of communicable diseases than the general population, and severe clinical outcomes when infected. The high turnover of people coming from the most disadvantaged segments of the population, together with the daily inflow/outflow of prison staff and facilities characterized by overcrowding, poor ventilation increase the risk of air-borne virus outbreaks. People living in prison are substantially more likely to experience drug-related problems than their peers in the community. Incarceration is associated with increases in blood-borne diseases-related risk behaviour among people who inject drugs. People living in prisons, because of the scarcity or lack of availability of needles, syringes and condoms, often share injecting equipment, tattooing and shaving materials, and practice unprotected sex. Individuals in contact with the criminal justice system often come from marginalized groups of society with a higher burden of poverty and discrimination, and with limited access to healthcare. Despite tailored preventive interventions should be implemented among vulnerable groups at the community level. Prisons can represent a point of access to integrated prison-community healthcare and social services. Delivering health protection and harm reduction programmes in prisons not only benefits the prison population but also has the potential to reduce the risk of transmission of some infectious diseases in the community, intervening earlier in the natural history of disease. The WHO has long supported the concept of prison health as an inseparable component of public health. However, a number of challenges hampers the successful implementation of such a concept, including the need for evidence-based decision making, inter-sectoral partnerships and adequate monitoring systems. Due to structural and operational reasons, conducting solid research and monitoring activities in prison settings is challenging and often available studies are mono-centric. This workshop will provide attendees with a comprehensive overview at European level of infectious diseases prevalence in prison populations and health services provided in detention facilities. The discussion of three European project collecting successful models of care for hepatitis elimination and implementation of vaccination services in prisons will create the context for an in-depth analysis of key challenges for prison health implementation and may help promote awareness that targeted interventions are feasible and effective in reducing infectious diseases burden among people living in prison and the community at large.

**Key messages:**

• Existing European initiatives contribute to building the evidence for tailored prevention & control interventions in prison settings.

• Adequate and effective prisons healthcare contribute to achieving the UN’s SDGs through improving health, reducing health inequalities and providing a fairer and safer society for all.

